# Obsessive-Compulsive, Psychotic, and Autism Dimensions Overlap in Real World: A Case Report

**DOI:** 10.1097/WNF.0000000000000561

**Published:** 2023-06-21

**Authors:** Mauro Scala, Laura Biondi, Alessandro Serretti, Chiara Fabbri

**Affiliations:** ∗ Department of Biomedical and Neuromotor Sciences, University of Bologna, Italy; † Institute of Psychiatry, Psychology and Neuroscience, King's College London, United Kingdom.

**Keywords:** obsession, stereotypy, schizophrenia, obsessive-compulsive disorder, autism spectrum disorders

## Abstract

Obsessions, compulsions, and stereotypes are common psychopathological manifestations of obsessive-compulsive, psychotic, and autism spectrum disorders (ASDs). These nosological entities may be present in comorbidity, with relevant clinical difficulties in the differential diagnosis process. Moreover, ASDs are a complex group of disorders, with a childhood onset, which also persist into adulthood and present heterogeneous symptom patterns that could be confused with psychotic disorders.

**Methods and Results:**

We report a case of a 21-year-old man characterized by sexual and doubt obsessions; disorganized, bizarre, and stereotyped behaviors and compulsions; and social withdrawal, inadequate social skills, visual dispersions, and hypersensitivity to light stimuli. Obsessive and compulsive features were initially included within the differential diagnosis of psychotic and obsessive-compulsive spectrum disorders. However, aforementioned psychopathological elements did not improve when multiple antipsychotic drugs (olanzapine, haloperidol, and lurasidone) were administered in the hypothesis of schizophrenia and even worsened with clozapine therapy at a dose of 100 mg/d. Obsessions and compulsions progressively reduced during the fluvoxamine 14-week treatment paradigm at a dose of 200 mg/d. Considering the persistent deficits in social communication and interactions as well as the restricted interests pattern, a differential diagnostic hypothesis of ASD was formulated, and it was then confirmed at the final evaluation at a third-level health care center.

**Conclusions:**

We discuss similarities and differences in the psychopathology of obsessions, compulsions, and stereotypes in the previously mentioned disorders, to underline factors that can help in the differential diagnosis of similar cases, and consequently in the appropriateness of treatment choice.

Obsessions and compulsions are commonly associated with obsessive-compulsive disorders (OCDs), a category of internalizing disorders, but they are also common features of different psychotic disorders (schizophrenia, schizotypal personality disorder [SPD]) and autism spectrum disorders (ASDs).^1^ This aspect may complicate the differential diagnosis process.

Whereas OCD and schizophrenia have typically an onset during adolescence or early adulthood,^2,3^ ASD manifests in early childhood and usually persists into adulthood, with repercussions on functioning and participation to daily activities.^4^

These disorders frequently manifest within a comorbidity framework. Prevalence estimates for obsessive and compulsive phenomena in the mentioned diseases are quite variable, as they are influenced by the clinical heterogeneity of these pathologies, including the broad spectrum of intellectual and verbal abilities. The prevalence rates of OCD in patients with schizophrenia vary between 3.8% and 59.2%,^5^ those of SPD in OCD vary from 5 to –50%,^6^ and finally, OCD is reported to occur as a comorbid condition in ASD in 37% of the cases^7–10^

It is often difficult to establish if certain individuals with ASD manifest a clinical picture that merits a co-occurring diagnosis of OCD. There is indeed an ongoing debate concerning the different nature of obsessions and repetitive behaviors in OCD and in ASD.^11,12^

In this regard, we present a case that can be useful to establish similarities and differences in the psychopathology of obsessions and compulsions across different disorders, to improve the precision and accuracy of the diagnostic approach in these patients.

## CASE PRESENTATION

P is a 21-year-old man of Chinese origin. He was born by cesarean delivery after a full-term pregnancy. As early as 3 years, P was described by his parents as not entirely responsive to human contact; he manifested certain stereotypes, such as staring intently at the sun or light sources and vocal stereotypes, such as the decontextualized repetition of his favorite words. In contrast, he never exhibited any motor stereotypes. During primary school, he faced some challenges in acquiring reading, writing, and calculating abilities principally because of concentration difficulties for background noise in classroom settings. When he was 12 years old, he moved to Italy with his family, because his father's gambling disorder had caused financial problems. Apart from his father, the family history of psychiatric disorders was negative. Nevertheless, P reported suffering from lack of sufficient parental attention. Because he began attending school in Italy, he acquired a basic knowledge of the Italian language, but he experienced difficulties in focusing, memory, and content learning (partly related to Italian language impairments) with relevant repercussions on his educational achievements. Moreover, during his initial years of education in Italy, P was victim of verbal and physical bullying by his peers. At 15 years of age, because of his rapid and significant social withdrawal, anhedonia, and apathy, he underwent a few visits at the community Services of Child Neuropsychiatry. Clinical evaluation and electroencephalogram led to no neuropsychiatric diagnosis.

The first visit to our outpatient Mental Health Centre was at the age of 18 years, as result of severe dietary restriction and physical exhaustion. However, no evidence of an eating disorder was found after clinical evaluation. This dietary restriction was indeed part of a generalized poor self-care, including poor personal hygiene. At that time, delusions or hallucinations were not observable. The prevalent psychopathological features were obsessions. They showed various types of content: sexual (sexual urges toward women, especially with incestuous content), blasphemous, aggressive (urgency to prick his own eye, let obscenities and impulses slip away), and of doubt (eg, uncertainty of stating things incorrectly or to not recall information). Associated compulsions resulted in insistently asking for repetition of sentences, for the name of medications taken, and for his diagnosis, despite understanding the content of the utterance. Furthermore, P exhibited negativism, long latency of response, sporadic echopraxia, slowed psychomotricity with figée facial expression, and little responsiveness to social interaction. He did not maintain eye contact with health care professionals, but he was fixated on facial or environmental details. His affectivity was restricted. Problems with short-term memory and fluctuations of attentional abilities emerged during interviews and were confirmed by the patient himself. During further evaluations, his conversations became awkward, and we observed a suspicious and distrustful attitude toward others outside the family. However, explicit ego boundary permeability with paranoid ideation was never present. The sensorium was characterized by elementary visual dispersions of changing intensity, with emotional involvement at times of stress. For this reason, he underwent an ophthalmological examination that did not reveal any eye abnormalities. P never showed any interest in the world around him except for a restricted and persistent interest in basketball. However, he preferred to spend up to 7 hours per day watching basketball videos on the Internet rather than playing with peers. Interaction with health professionals was unusual and inadequate (eg, he stood in front of the medical office door, he tried to enter it without knocking, he listened to music with headphones during the visit). Moreover, he lacked a clear perception of interpersonal space. He often presented forgetful, disorganized (eg, difficulties in planning a daily routine and in completing tasks), and unsafe behaviors (eg, cycling on the highway).

The first diagnosis that P received was schizophrenia, because of the presence of visual hallucinations (no auditory hallucinations were reported), disorganized speech and behavior, and negative symptoms.^13^ In this regard, we attributed adolescent maladaptive behaviors and social withdrawal to a prodromal symptom of a psychotic feature, and we considered migration as a severe environmental risk factor.^14^ Therefore, he was first treated with olanzapine titrated up to 20 mg daily for 3 months; then because of lack of efficacy, olanzapine was combined with haloperidol up to 5 mg for 2 months; and finally, we switched to lurasidone titrated up to 140 mg/d for other 3 months. Lack of clinical benefit was deduced by persistence of visual hallucinations, negative symptoms with social withdrawal, language abnormalities, and disorganized behaviors. Obsessions changed neither in content nor in frequency. Given these circumstances, a diagnosis of treatment resistant schizophrenia was made,^15^ and it was decided to start clozapine (25 mg/d), progressively increased up to 225 mg/d for 7 weeks. Initially, there was an increase in the hedonic drive, but there were no changes in terms of behavior. After 4 weeks, P manifested several adverse effects, including anhedonia, psychomotor slowing, worsening of ideo-behavioral disorganization, an increase in response latency times, sedation, and persistent hypersalivation. During the fifth week (when he was taking 100 mg/d), P developed bizarre compulsive behaviors, devoid of ideational content (counting the number of hairs on the arms and wrinkles on the fingers of the hands), with very little resistance to these compulsions. He also repeated obsessive sexual thoughts and questions to health care personnel. Especially in social circumstances, elementary visual dispersion (flashes of light, phosphenes, glares, luminous bodies) and sensitivity to light stimuli became more intense and frequent. The level of negativity, uncooperativeness, irritability, and dysphoria increased. During this period, P once again presented a drastic reduction in food and water intake in the absence of a clear conceptual content, intentionality, or awareness. The clinical team decided to discontinue clozapine gradually by 25 mg per week and to reevaluate the diagnosis. The frequency of obsessive ideas steadily decreased. In the hypothesis of a possible SPD, we used the Structured Clinical Interview for *DSM-5*—Clinical Version,^16^ which, however, did not reveal any personality disorder. The improvement of symptoms with the decalage of clozapine indicated that a psychotic disorder was very unlikely to underlie the obsessive-compulsive (OC) manifestations. In the diagnostic hypothesis of a severe OCD, fluvoxamine was started at 50 mg/d (clozapine had already been discontinued) and titrated up to 300 mg/d, as this drug could also have been beneficial for treating concomitant psychotic symptoms.^17^ At this dose, P developed irritability, insomnia, and overall increased activity; therefore, the dose was reduced to 200 mg/d, which was well tolerated. After 14 weeks of treatment, benefits on OC symptomatology were evident and remained stable thereafter. Behavior became more appropriate to the context. However, visual dispersions persisted especially in social contexts and during demanding tasks. Although verbal fluency improved, P continued not to benefit from the company of others. During all the time of observation, he showed poor insight of his condition.

**FIGURE 1:**
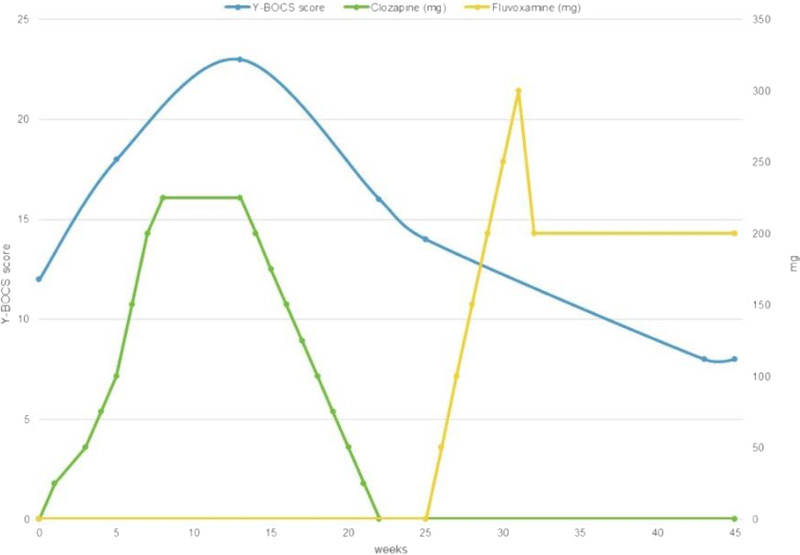
clozapine and fluvoxamine dose (*y* axis on the right) with Yale-Brown Obsessive Compulsive Scale scores (*y* axis on the left).

To investigate and quantify OC symptomatology and treatment response over time, the Yale-Brown Obsessive Compulsive Scale was used^18^ at 6 time points. As illustrated in Figure 1, OC symptoms worsened during the titration of clozapine, whereas they improved with fluvoxamine.

Despite the improvement during treatment with fluvoxamine, the diagnosis of OCD did not reflect well complexity of P's symptomatology. Consequently, a differential diagnostic hypothesis of ASD was formulated because P always presented with difficulties in social communication and interactions, considered to be more common in ASD then OCD,^12^ and with specific deficits in interpreting his own and others' mental states.^19^ Neurological abnormalities were excluded (electroencephalogram, brain nuclear magnetic resonance, and levels of vitamin B12/folate were normal). Further medical and psychological evaluations at a third-level health care center for ASD showed impairments in verbal and visual-spatial short- and long-term memory, as well as in executive and attentional functions. The Autism Diagnostic Observation Schedule—Second Edition and the Autism Diagnostic Interview—Revised, criterion standard assessment measures in the evaluation of ASD,^20^ confirmed ASD diagnosis together with the *Diagnostic and Statistical Manual of Mental Disorders* (Fifth Edition)—*Text Revision*,^13^ clinical interview.

## DISCUSSION

The present case report highlights the importance of exploring the psychopathological experience of obsessions and compulsions, differentiating them from other psychopathological elements, to avoid incorrect diagnoses.

In the case described, distinguishing symptoms of the psychotic spectrum from those of the OCD area was a challenging task. In the case of schizophrenia, it is important to differentiate obsessions from “thought insertions,” which are typical of early stages or relapses of the disease, and pathognomonic of permeability in the ego boundaries.^21^ Thought insertions are perceived as thoughts not genuinely originated from one's own mind, they are devoid of agency thinking, characterized by a reference or paranoid ideational content. In contrast, obsessions in schizophrenia are ego dystonic, characterized by a specific content (eg, contamination), associated with repetitive rituals and the capacity of agency thinking is maintained.^22,23^ In the case described, P manifested agency thinking, with sexual and doubt obsessions, initially embedded in a slightly psychotic background of paranoid attitude toward nonhousehold members. These symptoms were associated with repetitive and bizarre stereotypes (eg, intense observation of light sources). Obsessive-compulsive spectrum symptoms were unresponsive to various antipsychotics, and they worsened with clozapine. The relationship between clozapine, an antipsychotic with significant antiserotoninergic action, and the occurrence or worsening of OC symptoms and OCD is a well-known issue, as demonstrated by recent cross-sectional studies.^24,25^ This finding corroborated the OCD diagnostic hypothesis. However, the presence of OC symptoms in patients with schizophrenia is frequent; therefore, some authors have proposed a new clinical entity: the “schizo-obsessive disorder.”^26^ In comparison with schizophrenia, in this nosological entity, OC symptoms are typically more severe and manifest at earlier stages of the disease. They are associated with higher rates of anxiety and depression, and an increase in hospitalization risk.^27,28^ Obsessive-compulsive symptoms are also common features of SPD^29^ and result in a worse prognosis in this case as well.^30–32^ In the case of P, impaired interpersonal relationships were characterized by acute distress, unusual visual perceptual experiences, circumstantial and stereotyped thinking/language, inadequate behavior, and reduced affectivity. However, there was no concrete magical thinking, nor marked eccentricities and oddities, and a diagnosis of SPD was excluded. After 14 weeks of treatment with fluvoxamine at 200 mg/d, P's obsessions and ritualistic behaviors markedly decreased in frequency and intensity, despite visual dispersion remained unchanged. This outcome is in line with the results of a 12-week, double-blind, placebo-controlled trial^33^ that investigated the efficacy of fluvoxamine in the treatment of 30 adults with ASD. The drug was found to be superior to placebo in reducing obsessions and compulsions, maladaptive behaviors, and social withdrawal. When fluvoxamine 300 mg was prescribed, P developed irritability and insomnia, suggesting that patients with ASD may be more sensitive to the effects of fluvoxamine than patients with OCD. This is consistent with the increased risk of agitation induced by SSRIs observed in patients with ASD.^34^ Apart from OC symptomatology, stereotypes, hypersensitivity to light stimuli, and restricted interests, P always manifested a complete absence of social awareness, beliefs' recognition, emotional reciprocity, eye contact, and facial expressions interpretation, thus showing a clear impairment of theory of mind tasks.^19^ For these reasons, we have moved toward a possible diagnosis of ASD, later confirmed. Nevertheless, attempting to rule out a potential OCD in comorbidity was emphasized. Indeed, there is a marked difference between obsessions in OCD and ASD. Repetitive activities and obsessive ego-syntonic behaviors are typical of ASD and may represent comforting activities.^35^ In fact, P found relief in repeatedly watching the same videos about basketball. On the contrary, OC symptoms in OCD are typically ego dystonic and distressing. In our case, we considered OC symptoms as an epiphenomenon of ASD and not a separate diagnosis of OCD. In fact, P was not alarmed by obsessions, repetitive behaviors, and vocal stereotypes, and he was unable to report his internal feelings. Only light stimuli and visual dispersions caused him aversion and subsequent anxiety. It has been hypothesized that stereotyping occurs in ASD as a result of the individuals' attempts to cope with their abnormal sensory processing,^36^ which can include hyperresponsiveness and hyporesponsiveness as well as sensory overload,^37^ similar to schizophrenia neuropsychology.^38^ These alterations appear to have an important role in food and water intake, selection, and refusal. For example, children with ASD are more likely to have eating difficulties than their healthy counterparts.^39,40^ In our study, however, the stated dietary limitations were not related to a specific ideational content, nor did they meet the diagnostic criteria for a concurrent eating disorder. They might be the consequence of a reduced capacity, even as an adult, to care for oneself, and a decreased awareness of one's own physiological requirements, which were exacerbated by OC symptom intensification.

## CONCLUSIONS

This case report aims to emphasize the cross-diagnostical nature of OC symptoms, with fundamental implications for a correct diagnosis and treatment. Our considerations also underline the limitations of a categorical diagnostic approach, which is often not adequate to describe multidimensional phenomena.

Despite some apparent similarities in the manifestations of ASD and schizophrenia, from a psychopathological perspective, they may be considered as opposite conditions of a continuum, with highly self-oriented and mechanistic behavior and cognition in ASD whereas hypermentalistic cognitive style in schizophrenia.^41^

Another important point is the need of more information on the comorbidity between ASD and OCD, and other concomitant symptoms, such as eating problems. Developing and validating instruments to accurately evaluate OCD symptoms in ASD patients may be of great utility. Finally, finding effective treatments for obsessions and repetitive behaviors in patients with ASD is an important priority, as the potential efficacy of antidepressants such as fluvoxamine is still doubtful, particularly in the long term.
